# Prevalence and Reporting Rates of Extraspinal Findings for Lumbar Spine Magnetic Resonance Imaging in a Ghanaian Tertiary Hospital

**DOI:** 10.7759/cureus.82527

**Published:** 2025-04-18

**Authors:** Klenam Dzefi-Tettey, Emmanuel Kobina Mesi Edzie, Albert D Piersson, Edmund K Brakohiapa, Harold R Nixon, Emmanuel K Jackson, Kwasi O Armah, Ansumana S Bockarie, Henry Kusodzi, Abdul R Asemah

**Affiliations:** 1 Department of Radiology, School of Medicine, University of Health and Allied Sciences, Ho, GHA; 2 Department of Radiology, Korle-Bu Teaching Hospital, Accra, GHA; 3 Department of Medical Imaging, School of Medical Sciences, University of Cape Coast, Cape Coast, GHA; 4 Department of Diagnostic Imaging, University of Cape Coast, Cape Coast, GHA; 5 Department of Radiology, University of Ghana Medical School, Accra, GHA; 6 Department of Radiology, Ghana College of Physician and Surgeons, Accra, GHA; 7 Department of Internal Medicine, School of Medical Sciences, University of Cape Coast, Cape Coast, GHA

**Keywords:** extraspinal findings, ghana, lumbar spine, magnetic resonance imaging, prevalence rate

## Abstract

Background

Extraspinal findings are commonly detected on magnetic resonance imaging of the lumbar spine, but these findings are sometimes omitted from radiological reports. Failing to report these findings could have a clinical impact on the patients. The purpose of this study was to determine the prevalence and reporting rates of extraspinal findings on lumbar spine magnetic resonance imaging.

Methods

Retrospective analysis was done on lumbar spine magnetic resonance images done at the Korle-Bu Teaching Hospital between January 2020 and December 2021. A total of 1267 patients underwent lumbar spine magnetic resonance imaging within the period. The degree of clinical significance of the extraspinal findings was ascertained using the computed tomography colonography reporting and data system classification scheme. The reporting rate was determined by referring to the archived radiological reports. Statistical analysis was done using IBM SPSS Statistics for Windows, Version 25 (Released 2017; IBM Corp., Armonk, New York, United States).

Results

A total of 737 extraspinal findings were detected from 530 patients. The overall reporting rate of extraspinal findings was 62.6% (461/737). The most common extraspinal finding was a simple renal epithelial cyst (n = 333). Clinically significant findings were detected in 107 out of the 530 patients; 36.4% of the clinically significant findings were not reported when compared with the archived reports.

Conclusion

Extraspinal findings on lumbar spine imaging were common in our study population. When radiologists are reporting lumbar spine magnetic resonance imaging, it is crucial to be aware of the risk of missing clinically significant findings.

## Introduction

In today’s radiological practice, there is an increase in the reporting of incidental findings [1]. These incidental findings are commonly encountered as a result of an increase in the use of imaging examinations, many of which include magnetic resonance imaging (MRI) and computed tomography (CT) scans [2] together with the rapidly advancing digital evaluation of radiological images. Picture Archiving Communication System (PACS) is an important tool for digitizing and archiving radiological images in a more convenient and productive manner. The introduction of PACS has led to a significant increase in the number of reported incidental findings and suggested follow-up studies on patients [3].

Currently, MRI is the best imaging modality for evaluating the spine [4]. It is highly sensitive for detecting anatomic abnormalities and gives a good soft tissue resolution [5]. It may show incidental findings in the abdomen and pelvis, which may be clinically significant or may not have any clinical implications. Hence, careful evaluation of structures outside the region of interest may enable early detection of potentially life-threatening conditions [1].

Even though lumbar spine MRI examinations frequently reveal incidental findings, extraspinal findings are under-reported [6]. In this study, the term extraspinal finding was used to refer to any findings outside the lumbar spine. The aim of this study was to estimate the rates with which extraspinal findings were reported. We reported the prevalence of these extraspinal findings and highlighted the associated degree of clinical significance using the CT Colonography Reporting and Data System (C-RADS) classification [7]. The objective of this classification is to limit the reporting of unimportant findings in order to address issues relating to cost and burden of work-up, patient anxiety, and harm resulting from follow-up tests. We decided to investigate lumbar spine imaging because low back pain is one of the most common complaints in developing countries [8]. By reporting these findings and making an accurate diagnosis, radiologists may be able to prevent unnecessary investigations. On the contrary, failing to report these findings may raise clinical issues because it may have a significant impact on the patient’s life.

## Materials and methods

Design

This retrospective study was performed over a two-year period from January 2020 to December 2021. Retrospectively, we searched the entire digital database for patients who underwent lumbar spine MRI within this period. All lumbar spine MRI examinations were included except for repeated examinations. The Ethical Review Board of Korle-Bu Teaching Hospital approved this study, and it was conducted according to the Helsinki Declaration.

Image analysis

The MR images were reviewed independently by two experienced radiologists with more than 15 years of practice. They were both blinded to the archived reports of the images. After a consensus decision was reached by the two radiologists, the findings were categorized into reported (seen in the archived reports) and unreported (not seen or missed in the archived reports) from which the reporting rate (reported extraspinal findings/total extraspinal findings [reported + unreported]) were obtained as percentages. The data was then entered into a structured database created on Microsoft Excel according to the specific organs involved. Patient demographic data (age and gender) were recorded as well. 

Due to the differences in the classification systems reported in the literature, we employed the C-RADS classification scheme in grading the abnormalities detected. All anatomic variants were assigned C-RADS E1. Clinical findings where no work-up was required were assigned to C-RADS E2. C-RADS E3 was assigned to findings that were likely clinically insignificant or likely benign; hence, further work-up could be performed if indicated. Finally, C-RADS E4 was assigned to findings that were clinically significant requiring further work-up [9].

MRI protocol

The MRI examinations were carried out on a 1.5 Tesla Toshiba Vintage Titan MRI machine with serial number GH-0029-01-CMR-01 (Toshiba Medical Systems Corporation, Otawara, Japan) using a surface coil, and patients were scanned in supine position. A routine lumbar spine MRI protocol was used: 4 mm sagittal T1-weighted (T1W), fluid attenuation inversion recovery (FLAIR) or T1-weighted, T2-weighted, short Tau inversion recovery (STIR), stacked or contiguous axial T2W and axial T1W turbo spin-echo sequences. Most of the subjects were scanned without intravenous (IV) gadolinium; however if infectious, inflammatory, or neoplastic etiologies were suspected, then contrast-enhanced MRI with IV gadolinium was performed.

Statistical analysis

Descriptive statistics was used to summarize all extraspinal findings in the form of count and percentages. Mean age comparison between men and women was done using Student's independent sample t-test. The chi-square test for independence was used for measuring the association between the various categories of the C-RADS classification scheme and gender. Statistical significance was set at P ≤ 0.05. Statistical analysis was done using IBM SPSS Statistics for Windows, Version 25 (Released 2017; IBM Corp., Armonk, New York, United States).

Ethical considerations

Ethical approval for this study was obtained from the Korle-Bu Teaching Hospital institutional review board with clearance number KBTH-ADM/0043/2024. This study conformed to the 1975 Helsinki Declaration.

## Results

Extraspinal findings were found in 530 out of 1267 patients who underwent lumbar spine MRI examination within the period, with a minimum age of four years and a maximum age of 96 years. About 737 extraspinal findings were reported in 530 patients (Table 1). The overall reporting rate of extraspinal findings was 62.6% (461/737). Out of the 530 patients, 173 (32.6%) were male patients and 357 (67.4%) were female patients. The mean age of our study population was 54.15 (17.298) years. There was a statistically significant difference between the mean ages of male patients and female patients detected with extraspinal findings (P < 0.001).

**Table 1:** Summary of 737 extraspinal findings and organs involved

Extraspinal findings	Related organ	Frequency (%)	Reported	Unreported	Reporting rate (%)
Hydronephrosis	Kidney	32 (4.3)	24	8	75.0
Simple renal epithelial cyst		333 (45.0)	254	79	76.3
Parapelvic cyst		2 (0.3)	2	–	100.0
Extrarenal pelvis		12 (1.6)	1	11	8.3
Renal atrophy		8 (1.1)	6	2	75.0
Ectopic kidney		1 (0.1)	1	–	100.0
Malrotated kidney		3 (0.4)	–	3	0.0
Complex renal cyst		1 (0.1)	–	1	0.0
Prominent renal dromedary hump		2 (0.3)	–	2	0.0
Complex cystic mass		2 (0.3)	–	2	0.0
Uterine fibroids	Uterus	169 (22.9)	95	74	56.2
Endometrial cavity fluid		2 (0.3)	2	–	100.0
Fluid in pouch of Douglas		7 (0.9)	7	–	100.0
Retroverted uterus		32 (4.3)	1	31	3.1
Endometrial cyst		6 (0.8)	6	–	100.0
Absent uterus		6 (0.8)	–	6	0.0
Anteflexed uterus		2 (0.3)	–	2	0.0
Thickened endometrium		1 (0.1)	–	1	0.0
Diffuse adenomyosis		2 (0.3)	–	2	0.0
Adnexal mass	Ovary	1 (0.1)	1	–	100.0
Ovarian cyst		39 (5.3)	23	16	59.0
Solid ovarian mass		5 (0.7)	5	–	100.0
Bladder diverticuli	Urinary bladder	18 (2.4)	13	5	72.2
Bladder trabeculations		3 (0.4)	1	2	33.3
Gall bladder hydrops	Gall bladder	2 (0.3)	–	2	0.0
Gall stone		2 (0.3)	–	2	0.0
Fusion of T10/T11	Thoracic spine	2 (0.3)	–	2	0.0
Hemangioma at T10		2 (0.3)	–	2	0.0
Pelvic ascites	Pelvis	8 (1.1)	6	2	75.0
Enlarged prostate	Prostate	11 (1.5)	7	4	63.6
Fecal impaction	Rectum	7 (0.9)	3	4	42.9
Simple hepatic cyst	Liver	1 (0.1)	1	–	100.0
Muscle atrophy	Gluteus maximus	1 (0.1)	1	–	100.0
Skin nodules	Skin	2 (0.3)	–	2	0.0
Psoas abscess	Psoas muscle	3 (0.4)	–	3	0.0
Retroperitoneal mass	Retroperitoneum	4 (0.5)	1	3	25.0
Seroma right breast	Breast	1 (0.1)	–	1	0.0
Anterior abdominal defect	Abdominal wall	1 (0.1)	–	1	0.0
Intramuscular lipoma	Erector spinae muscle	1 (0.1)	–	1	0.0
Total		737	461	276	62.6%

The extraspinal findings involved 17 different organs including the liver, prostate, gall bladder, psoas, and retroperitoneum, among others. However, the kidney, uterus, ovary, and urinary bladder were the most involved organs representing 388/737 (52.6%), 227/737 (30.8%), 45/737 (6.1%), and 21/737 (2.8%), respectively. Among all the extraspinal findings, a simple renal epithelial cyst was the most common, representing 333/737 (45.0%); all other findings represented the remaining 55.0% (Table 1). 

Normal variants were classified as C-RADS E1 and were found in 4/530 patients (two each in both male patients and female patients). The largest portion of extraspinal findings was classified in the C-RADS E2 category and were found in 379/530 patients (137 in male patients and 242 in female patients). The most prevalent finding in this category was a simple renal epithelial cyst, representing 76.3% (254/333). We detected 32 patients with retroverted uterus and 12 patients with extrarenal pelvises but only one of each finding was reported (Table 2 and Table 3).

**Table 2:** Classification of 737 extraspinal findings based on C-RADS C-RADS: CT Colonography Reporting and Data System

	Extraspinal finding	Reported	Unreported	Non–detection rate (%)
E1: Normal variant				
	Anteflexed uterus	–	2	100.0
	Prominent renal dromedary hump	–	2	100.0
E2: Likely insignificant findings				
	Renal cyst	254	79	23.7
	Parapelvic cyst	2	–	0.0
	Extrarenal pelvis	1	11	91.7
	Retroverted uterus	1	31	96.9
E3: Likely significant findings				
	Uterine fibroids	95	74	43.8
	Endometrial cavity fluid	2	–	0.0
	Fluid in the pouch of Douglas	7	–	0.0
	Ovarian cyst	23	16	41.0
	Endometrial cyst	6	–	0.0
	Fecal impaction	3	4	57.1
	Simple hepatic cyst	1	–	0.0
	Absent uterus	–	6	100.0
	Malrotated kidney	–	3	100.0
	Hemangioma at T10	–	2	100.0
	Gall stone	–	2	100.0
	Thickened endometrium	–	1	100.0
	Intramuscular lipoma	–	1	100.0
E4: Clinically significant findings				
	Hydronephrosis	24	8	25.0
	Adnexal mass	1	–	0.0
	Bladder diverticular	13	5	27.8
	Bladder trabeculations	1	2	66.7
	Pelvic ascites	6	2	25.0
	Enlarged prostate	7	4	36.4
	Renal atrophy	6	2	25.0
	Solid ovarian mass	5	–	0.0
	Atrophy of the gluteus maximus	1	–	0.0
	Seroma right breast	–	1	100.0
	Ectopic kidney	1	–	0.0
	Fusion of T10/T11	2	–	0.0
	Skin nodules	–	2	100.0
	Psoas abscess	–	3	100.0
	Retroperitoneal mass	1	3	75.0
	Gall bladder hydrops	–	2	100.0
	Anterior abdominal wall defect	–	1	100.0
	Complex renal cyst	–	1	100.0
	Diffuse adenomyosis	–	2	100.0
	Complex cystic mass	–	2	100.0

**Table 3:** Comparison of C-RADS distribution and mean age across gender ^a^Statistically significant; ^b^Not statistically significant C-RADS: CT Colonography Reporting and Data System

	Total (N = 530)	Male (N = 173)(%)	Female (N = 357)(%)	P–value
Age (mean ± SD)	54.15 ± 17.298	59.39 ± 16.71	51.61 ± 17.026	< 0.001^a^
C-RADS E SCORE				
E1	4(100.0)	2(50.0)	2(50.0)	0.874^b^
E2	379(100.0)	137(36.1)	242(63.9)	0.021^a^
E3	247(100.0)	16(6.5)	231(93.5)	< 0.001^a^
E4	107(100.0)	69(64.5)	38(35.5)	0.381^b^

**Figure 1:**
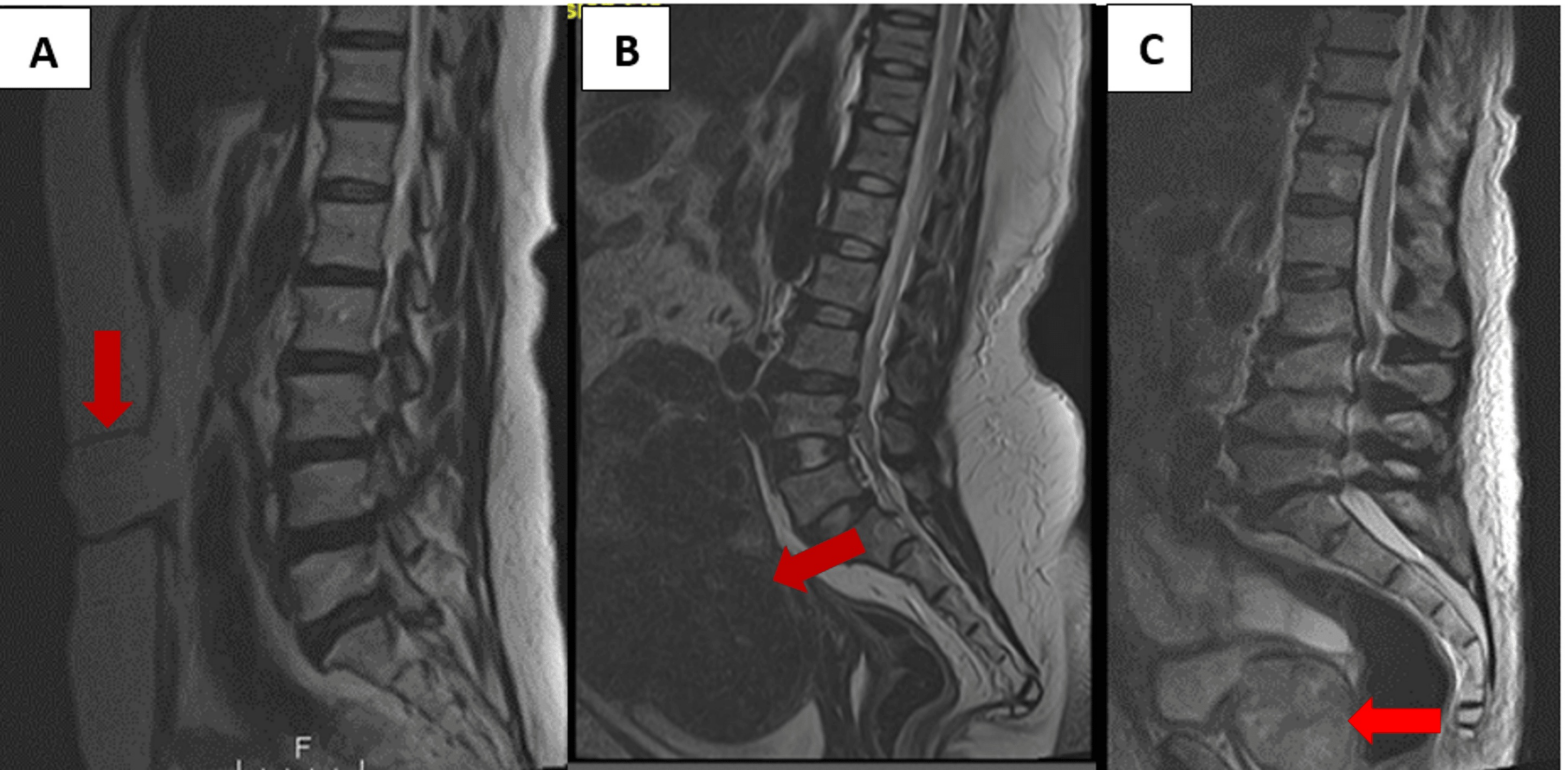
(A) Unenhanced sagittal T2W MR image of the lumbosacral spine of a 52-year-old man showing an anterior abdominal wall defect. (B) Unenhanced sagittal T2W MR image of the lumbosacral spine of a 40-year-old woman showing an enlarged anteverted uterus with multiple uterine fibroids of varying sizes and (C) unenhanced sagittal T2W MR image of the lumbosacral spine of a 65-year-old man showing an enlarged heterogeneous prostate gland. All depicted with the red arrows.

**Figure 2:**
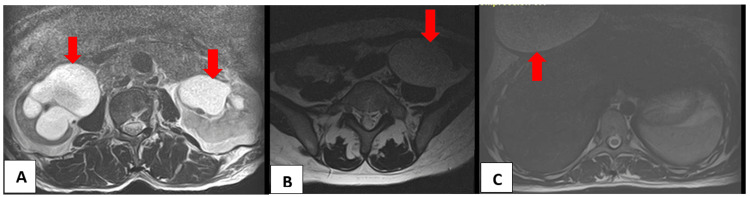
(A) Unenhanced axial T2W MR image of the lumbosacral spine of a 38-year-old man showing bilateral hydronephrosis worse on the right with significant renal cortical thinning. (B) Unenhanced axial T2W MR image of the lumbosacral spine of a 46-year-old woman showing a simple left ovarian cyst. (C) Unenhanced axial T2W MR image at the upper lumbar spine of a 41-year-old woman showing seroma, right breast. All depicted with the red arrows.

Figures 1, 2 show some of the extraspinal findings on the lumbar spine MRI of our patients.

Clinically significant findings represented as C-RADS E4 were detected in 107/530 patients. There was no statistically significant difference in clinically significant findings found in male patients and female patients (P = 0.381) (Table 3). Hydronephrosis constituted the majority of cases found to be clinically significant. A total of 32 cases of hydronephrosis were detected; however, eight (25.0%) of such cases were not reported. Other clinically significant findings such as psoas abscess, skin nodules, gall bladder hydrops, diffuse adenomyosis, and complex cystic mass were detected in more than one patient but none were reported (Table 2).

## Discussion

MRI of the lumbar spine can incidentally detect extraspinal findings which may or may not be clinically significant [10]. It is important to note that an incidental extraspinal finding occasionally may be more important than the presumed disease that triggered imaging. However, the clinical significance of extraspinal findings on patient health outcomes is not certain, and discussion remains ongoing. In the general population, extraspinal abnormalities on lumbar spine MRIs are widespread [6], but radiologists rarely report all extraspinal findings. In a total of 1,267 cases, we found that the prevalence of extraspinal findings was 42.2%. Prevalence rates greater than 40.0% have been reported in several other investigations [6,9,11-14]. In six independent studies that evaluated the use of MRI scans to identify incidental extraspinal findings of the lumbar spine, it was found that the prevalence rate was less than 30.0% in the target population. The quoted rates were as follows: 14.0%, 16.4%, 16.6%, 18.8%, 23.7%, and 29.0%, respectively [15-20]. Sizes of uterine fibroids, renal cysts, and ovarian cysts measuring 1-1.5 cm were all excluded from these studies. Studies that found a prevalence rate of more than 40.0% reportedly took into account all cystic lesions and fibroids, irrespective of size [9,11-14], similar to how the cases were recruited into our study. 

According to the study by Quattrocchi et al. [9], more than half (68.7%, 2060 out of 3000 patients) of the study group had extraspinal abnormalities on lumbar spine MRI. This high prevalence rate was mostly due to the MRI protocol used, which had a wider field of view (FOV). When identifying extraspinal lesions, the dimensions of the precise anatomical regions used in scan protocols are very important. It is generally known that the modification of the reconstructed FOV has a significant influence on image quality and ultimately diagnosis [21]. The intervertebral disc and its relationship to the neural structures are said to be more accurately and thoroughly assessed when the MR images are of high quality. Like MRI, the impact of large FOV in determining the prevalence of extraspinal findings is no different on CT scans. The frequency of extraspinal abnormalities on lumbar spine CT scans was assessed in two investigations. One study evaluated full FOV lumbar spine CT images and found the prevalence of extraspinal findings to be 40.5% [22]. Only 1.45% of extraspinal findings were found in the second study that examined lumbar spine CT images with a restricted FOV [23]. The wide spectrum of reported prevalence in the literature and our study is likely due to differences in FOV and the use of large cohorts (≥ 3000 cases of lumbar spine) in some of the studies. 

The spectrum of extraspinal findings from a large cohort (4250 patients) is reflected in the range of extraspinal findings (1509 findings) reported by Khasawneh et al. [11]. These authors found two intramuscular metastatic deposits from lung cancer in two different patients. Their study also found two cases of appendiceal mucocele and a case of dilated bowel loops with intussusceptions occurring in the small bowel. Quattrocchi et al. also reported two cases of intramuscular metastatic deposits from lung cancer, five cases of appendiceal mucocele, and three cases of small bowel dilatation secondary to intussusception (from 2060 findings) [9]. On the other hand, none of such cases were detected in our study. This variation may be caused by the differences in the population size investigated and also could be due to the sparing use of MRI in our jurisdiction.

Most of the study's findings were categorized as C-RADS E2 (Table 3). A simple renal epithelial cyst was the most prevalent finding, accounting for 87.9% (333/379) of C-RADS E2 and 45.2% (333/737) of all extraspinal abnormalities. A simple renal epithelial cyst can develop in normal kidneys with a rise in prevalence with advancing age [24]. These cysts were mostly multiple (n = 232; 69.7%) and more prominent on the left kidney. This was commonly observed in men in a ratio of 3:1. Our findings were consistent with Mensal et al. [25] and Gameraddin et al. [26] in terms of the male-to-female ratio. However, our ratio was different from what Carrim et al. [27] and Chang et al. [28] reported. Both reported a 4:1 male-to-female ratio. 

C-RADS E3 and C-RADS E4 lesions, which made up 33.5% (247/737) and 14.5% (107/737) of the total extraspinal findings respectively, have more relevance to radiologists interpreting lumbar spine MRI. This is substantially different from the 10.5% C-RADS E3 findings and 4.3% C-RADS E4 findings reported by Lee et al [22]. Similar findings of 31.1% C-RADS E3 and 12.9% C-RADS E4 were reported by Quattrocchi et al [9]. Hydronephrosis constituted majority of the clinically significant findings but represented only 4.3% of the extraspinal findings reported. According to other investigations, the prevalence of hydronephrosis varied between 0.2 and 12.2% [9,11,18,29]. Similar to earlier studies, hydronephrosis was considered a clinically significant finding [9,11,18] whilst other studies classified hydronephrosis as a likely clinically nonrelevant finding [29]. Although the cause of hydronephrosis may not be defined on lumbar MR images, its presence may help the practitioner find and address the underlying issue before kidney function is irreparably lost.

The reporting rate of extraspinal findings in this study was 62.6%, though many clinically significant findings were unreported. The reporting rate found in our study was greater than what was seen in other studies, ranging from 7% to 47% [9,11,18,30] but similar to what was reported by Semaan et al. (60%) [29]. We noted that findings in organs closer to the lumbar spine were highly reported (Table 1). This may indicate that the radiologists' primary areas of interest in reporting lumbar spine are spinal pathologies and its immediate structures. A non-detection rate of 36.4% was obtained when we discovered that 39 out of 107 C-RADS E4 lesions were missing from the archived reports. The non-detection rate of the clinically significant findings was less than that reported by Quattrocchi et al. (85.0%) [9] and Khasawneh et al. (58.8%) [11] but closer to what was reported by Semaan et al. (38.6%) [29]. The non-detection rate of extraspinal abnormalities among radiologists may vary depending on the practice norms at various facilities. We observed that whilst the non-detection rates for the various C-RADS categories in our study did not indicate any particular order, the study by Seeman et al. revealed that non-detection rates reduced as C-RADS category increased [29]. We made a hypothetical claim that perhaps radiologists from their study facility were reporting findings in a selective manner based on their clinical significance.

The non-detection rate of extraspinal lesions among radiologists may be caused by a number of circumstances in addition to those already discussed. These could include the radiologist's experience, the workload, the absence of detailed clinical information at the time of reporting, among others. Furthermore, the non-detection rate of extraspinal lesions may also be explained by a common diagnostic error known as Satisfaction of Search (SOS) and Perceptual Capture of Attention. According to studies, an early lesion or abnormality detected by the reading radiologist may divert exact cognitive focus, resulting in perceptual errors [30]. Once the radiologist is satisfied with the initial detection of an abnormality, the radiologist may end the case prematurely, and other lesions may remain undetected.

To reduce the incidence of non-detected extraspinal lesions, we suggest that when radiologists are reporting lumbar spine MR images, they should thoroughly evaluate all regions outside the spine in the FOV. However, reporting benign findings that are not clinically significant may cause some form of anxiety in patients and confuse some clinicians. In order to prevent needless procedures, we suggest that all extraspinal abnormalities should be mentioned in the radiological report with additional explanation of their clinical significance. 

One major limitation of our study was the absence of patient follow-up, which made it difficult to determine the sequelae of clinically significant extraspinal findings detected. Other limitations such as differences in MRI strengths and protocols across different institutions and differences in populations studied as well as the relatively small sample size used in our study may limit the generalizability of our findings. Further studies may be required to assess the relationship between reporting rates, under-reporting rates, extraspinal findings, and gender. 

## Conclusions

This study emphasized the clinical importance of extraspinal findings and the need for radiologists to carefully review the extraspinal regions. In order to reduce potential harm to patients, all extraspinal findings should be stated in the radiological reports and communicated in a manner that does not trigger patient’s anxiety and at the same time provide clues to physicians on the relevance of these incidental findings in relation to the patient’s health. 
